# Interleukin-16 is increased in obesity and alters adipogenesis and inflammation *in vitro*

**DOI:** 10.3389/fendo.2024.1346317

**Published:** 2024-03-13

**Authors:** Marjorie Reyes-Farias, Pablo Fernández-García, Patricia Corrales, Lorena González, Andrea Soria-Gondek, Ester Martínez, Silvia Pellitero, Jordi Tarascó, Pau Moreno, Lauro Sumoy, Gema Medina-Gómez, David Sánchez-Infantes, Laura Herrero

**Affiliations:** ^1^ Endocrinology department, Germans Trias i Pujol Research Institute (IGTP), Badalona, Spain; ^2^ Department of Biochemistry and Physiology, School of Pharmacy and Food Sciences, Institute of Biomedicine of the University of Barcelona (IBUB), Universitat de Barcelona (UB), Barcelona, Spain; ^3^ Department of Basic Health Sciences, University Rey Juan Carlos (URJC), Madrid, Spain; ^4^ Pediatric Surgery Department, Hospital Universitari Germans Trias i Pujol, Badalona, Spain; ^5^ Endocrinology and Nutrition Department, Institute Research and Hospital Germans Trias i Pujol, Universitat Autònoma de Barcelona, Barcelona, Spain; ^6^ General Surgery Department, Hospital Universitari Germans Trias i Pujol, Badalona, Spain; ^7^ Centro de Investigación Biomédica en Red de Fisiopatología de la Obesidad y Nutrición (CIBEROBN), Instituto de Salud Carlos III, Madrid, Spain

**Keywords:** obesity, immunometabolism, IL-16, inflammation, adipocytes

## Abstract

**Introduction:**

Obesity is a chronic condition associated with low-grade inflammation mainly due to immune cell infiltration of white adipose tissue (WAT). WAT is distributed into two main depots: subcutaneous WAT (sWAT) and visceral WAT (vWAT), each with different biochemical features and metabolic roles. Proinflammatory cytokines including interleukin (IL)-16 are secreted by both adipocytes and infiltrated immune cells to upregulate inflammation. IL-16 has been widely studied in the peripheral proinflammatory immune response; however, little is known about its role in adipocytes in the context of obesity.

**Aim & Methods:**

We aimed to study the levels of IL-16 in WAT derived from sWAT and vWAT depots of humans with obesity and the role of this cytokine in palmitate-exposed 3T3-L1 adipocytes.

**Results:**

The results demonstrated that IL-16 expression was higher in vWAT compared with sWAT in individuals with obesity. In addition, IL-16 serum levels were higher in patients with obesity compared with normal-weight individuals, increased at 6 months after bariatric surgery, and at 12 months after surgery decreased to levels similar to before the intervention. Our in vitro models showed that IL-16 could modulate markers of adipogenesis (Pref1), lipid metabolism (Plin1, Cd36, and Glut4), fibrosis (Hif1a, Col4a, Col6a, and Vegf), and inflammatory signaling (IL6) during adipogenesis and in mature adipocytes. In addition, lipid accumulation and glycerol release assays suggested lipolysis alteration.

**Discussion:**

Our results suggest a potential role of IL-16 in adipogenesis, lipid and glucose homeostasis, fibrosis, and inflammation in an obesity context.

## 1 Introduction

Obesity is a chronic, relapsing, and progressive disease with multifactorial etiology (1, 2). The worldwide incidence of this obesity has tripled in the last four decades, becoming one of the most prevalent diseases worldwide (3). It is characterized by an excessive or abnormal accumulation of adipose tissue in the body, caused by a long-term energy imbalance between calorie intake and expenditure (4). Obesity is also a significant risk factor for several chronic diseases, such as cardiovascular disease (CVD), insulin resistance (IR), type 2 diabetes (T2D), non-alcoholic fatty liver disease, some types of cancer, and reduced life expectancy (5–9).

White adipose tissue (WAT) is mainly composed of adipocytes, fibroblasts, and immune cells, such as macrophages, T and B cells, and mast cells (10). WAT is not only a depot for lipid storage but also an endocrine organ responding to metabolic changes. It is distributed into two main depots: subcutaneous (sWAT) and visceral (vWAT) (11, 12). In obesity, WAT expansion and dysfunction are accompanied by immune infiltration (13, 14). IL-16 is a proinflammatory cytokine that acts as a leukocyte chemoattractant factor and is secreted by several immune cell populations (15). The IL-16 pro-molecule is constitutive in T cells, mast cells, eosinophils, epithelial cells, fibroblasts, and dendritic cells (15), and requires processing and activation by caspase-3 cleavage, which releases the biologically active form of IL-16 (16). Using our data from RNAseq of WAT T lymphocytes from individuals with obesity, an upregulation of IL-16 was observed in enriched pathways related to SARS-CoV-2 infection, poor prognosis, and severe symptoms. The function of IL-16 in the peripheral proinflammatory immune response has been widely studied (15, 17–20), however, its role in adipocytes in the context of obesity is unclear. Therefore, we aimed to study the role of IL-16 in obesity, evaluating its effect on adipocyte biology. *IL-16* gene expression was elevated in vWAT from individuals with obesity and correlated with inflammatory markers. Moreover, *in vitro* studies demonstrated that treatment with recombinant IL-16 decreased the expression of genes related to inflammation, lipid metabolism, and adipose tissue remodeling. Finally, IL-16 blunted palmitate-induced lipid accumulation in 3T3-L1 adipocytes.

## 2 Methods

### 2.1 Study participants

The study was approved by the Ethical Committee of the Hospital Germans Trias i Pujol (Badalona, Spain) and followed the guidelines of the Declaration of Helsinki. Participants gave written informed consent prior to clinical data collection.

The cohort included 27 individuals: 13 with severe obesity and 14 with normal weight (**Table 1**). All individuals were evaluated between October, 2015 and September, 2021 by the same endocrinologist (S.P.), who followed the institution’s protocol for bariatric surgery (BS), according to BS criteria (Spanish Position Statement between Obesity, Endocrinology, Diabetes, and Surgery Societies). vWAT and sWAT biopsies were taken from individuals with severe obesity during BS and from individuals with normal weight at the time of consultation/minor surgery, mainly cholecystectomy and biopsies that were finally negative for tumors. Exclusion criteria were having active infectious or inflammatory pathologies other than those related to obesity, treatment with immunosuppressant drugs, or suffering from other forms of immunosuppression.

**Table 1:** Clinical parameters of the cohort of patients.

	Control (n = 14)	Obesity (n = 13)	P value
	Mean ± SD	Mean ± SD	
**Gender (male/females)**	0/14	0/13	
**Age (years)**	48.6 ± 8.2	46.2 ± 10.1	ns
**Weight (kg)**	64.9 ± 9.40	112.2 ± 12.0	*P*< 0.0001
**BMI (kg/m^2^)**	24.7 ± 2.5	43.5 ± 3.9	*P*< 0.0001
**Glucose (mg/dL)**	92.7 ± 15.4	106.5 ± 23.5	ns
**Insulin (mIU/L)**	5.4 ± 1.3	16.5 ± 11.8	ns
**HbA1c (%)**	4.8 ± 0.8	5.3 ± 1.2	*P*< 0.01
**HOMA**–**IR (%)**	1.3 ± 0.4	4.8 ± 4.2	ns
**Triglycerides (mg/dL)**	72 ± 28	126 ± 34	*P*< 0.01
**LDL-c (mg/dL)**	108 ± 31	91 ± 14	ns
**HDL-c (mg/dL)**	68 ± 9	42 ± 8	*P*< 0.0001
**Total cholesterol (mg/dL)**	190 ± 34	158 ± 18	*P*< 0.05

The study cohort included 14 controls and 13 patients with severe obesity undergoing bariatric surgery (all women). BMI, body mass index; HbA1c, glycated hemoglobin, HOMA–IR, homeostatic model of insulin resistance; LDL-c, low-density lipoprotein cholesterol; HDL-c, high-density lipoprotein cholesterol. Differences between controls and patients with severe obesity were assessed using Student’s t-test (normally-distributed) or Mann-Whitney test (nonnormally-distributed) for unpaired data. Normality was checked using the Shapiro–Wilk test. ns, not statistically significant, controls vs. patients with severe obesity.

### 2.2 Human serological analysis

Serum samples were collected after 12 h fasting on the day of surgery and 6 and 12 months afterward, and frozen at -20° C. An ELISA assay was performed at baseline, 6, and 12 months after surgery (R&D system Bio-Techne D1600). Glucose and insulin levels, and lipid profiles [total cholesterol, high-density lipoprotein (HDL) cholesterol, low-density lipoprotein cholesterol, and triglycerides], were measured in our certified core clinical laboratory by enzymatic methods.

Homeostatic model assessment of insulin resistance (HOMA–IR) was calculated as:


$$HOMA-IR=\frac{\left[Glu\cos e\frac{mg}{dL}\right]\times \left[Insulin\frac{m.u.int}{dL}\right]}{405}.$$

### 2.3 Adipose tissue collection and RNA isolation and processing

Fresh WAT samples collected during surgeries were transferred to liquid nitrogen and then frozen at –80° C. Total RNA was extracted from whole adipose samples using a standard column-affinity-based methodology (NucleoSpin RNA II; Macherey-Nagel). Next, 500 ng of total RNA was retrotranscribed into cDNA using random hexamer primers and Multiscribe reverse transcriptase (TaqMan reverse transcription reagents, ThermoFisher Scientific) according to the manufacturer’s instructions. Platinum Quantitative PCR SuperMix-UDG with ROX reagent (ThemoFisher Scientific) was used as the master mix reagent and the expression levels of each gene of interest were assessed using specific TaqMan probes Hs00913644_m1 for *IL-16* and Hs04194521_s1 for the housekeeping *Ppia* gene (ThermoFisher). Gene expression was calculated using the 2^-ΔΔCt^ method.

### 2.4 Cell culture experiments

All procedures in the cell culture room were performed under a laminar flow hood. Cells were grown at 37°C with an atmosphere of 95% air, 5% CO2. All reagents used during cell culture procedures were heated to 37°C in a water bath before use.

The 3T3-L1 murine preadipocyte cell line (CL-173, ATCC) was used between passages 13 and 15.

To obtain mature adipocytes, 3T3L1 preadipocytes were plated into 12-well plates and grown to 100% confluency, with the growth medium being replaced every other day. Two days after 100% confluency was reached, an induction medium was added (day 0 of induction for the differentiation period), and cells were incubated for 48 h (day 2 of the differentiation period). The induction medium was then changed for a differentiation medium (**Supplementary Table S1**), and cells were incubated for an additional 48 h (day 4 of the differentiation period). The differentiation medium was then changed to a maintenance medium (**Supplementary Table S1**) until the cells acquired a mature adipocyte phenotype (day 9 of the differentiation period). IL-16 treatment was performed using recombinant mouse IL-16 protein (Invitrogen, RP-8610) at a concentration of 1 or 10 ng/mL during 3T3-L1 differentiation to evaluate the effect of this cytokine during *in vitro* adipogenesis. Differentiated adipocytes were treated for 24 h with the same concentrations of IL-16.

Fatty acid (FA) treatment: Sodium palmitate (Sigma-Aldrich, P9767) was conjugated with FA-free BSA at a 5:1 ratio (21). Mature adipocytes were incubated with 1 mM of this solution for 24 h. The control group was incubated with 0.1% BSA.

Free glycerol was measured using a Serum Triglyceride Determination Kit (Catalog Number TR0100, Sigma-Aldrich, USA).

### 2.5 Oil red O staining of 3T3-L1 preadipocytes

Lipid accumulation was evaluated in 3T3-L1 cells by staining with Oil Red O (Sigma Aldrich, O0625). The stock solution was prepared by adding 300 mg of Oil Red O powder (Sigma Aldrich, O0625) to 100 ml of 2-propanol (Alfa Aesar, 36644). The solution was protected from light and stirred overnight. The final solution was filtered through Whatman paper to remove dye precipitates. Oil Red O working solution was prepared by mixing three parts of the Oil Red O stock solution with two parts of distilled water, incubated for 10 minutes at room temperature protected from light, and filtered through a syringe filter unit (Merck, SLHV033RB). Plated cells were carefully washed once with PBS, avoiding disruption of the cell monolayer. Next, cells were fixed with 500 μL of 4% paraformaldehyde (Sigma-Aldrich, 47608) diluted in PBS, and incubated for 30 min at room temperature. Once the cell monolayer was fixed, it was washed twice with distilled water and then incubated for 5 min with 500 μL of 60% isopropanol. Next, 500 μL of Oil Red O working solution was added and incubated for 15 min to stain intracellular lipids. The staining solution was removed and the cell monolayer was washed three times with distilled water. The cell monolayer was photographed using a microscope (Leica DM IL LED, Leica Biosystems) to assess the lipid vesicles in mature adipocytes. Staining was quantified by extracting the stain with 1 mL of 100% isopropanol, and 200 μL of this solution was placed in a 96-well plate (Greiner Bio-one, 655101) to measure the absorbance at 492 nm using a Varioskan LUX Multimode Microplate Reader (Thermo Scientific).

### 2.6 Cell RNA extraction and quantification

RNA was extracted from cultured cells using Trizol™ reagent (Invitrogen, 15596026) according to the manufacturer’s instructions. RNA was precipitated using GlycoBlue® (ThermoFisher, AM9515) in the isopropanol step. Samples were then left overnight at -20°C and were processed the following day according to the manufacturer’s protocol. The RNA pellet was resuspended in 30 µL of nuclease-free water and quantified with Nanodrop. Next, 1 µg of total RNA was retrotranscribed using M-MLV reverse transcriptase (Invitrogen, 28025-0113) according to the manufacturer’s instructions. A LightCycler 480 SYBR Green I Master (Roche, 4887352001) was used as the master mix reagent, and expression levels of the genes of interest were assessed using specific primers (**Supplementary Table S2**). Gene expression was calculated using the 2^-ΔΔCt^ method, with *Ppia* as the housekeeping gene, and expressed as arbitrary units.

### 2.7 Statistical analysis

A public database generated by our laboratory (GEO repository: GSE236145) containing RNAseq from human WAT-infiltrated T cells was used to decipher the potentially relevant role of IL-16 in pathways related to immunometabolism in obesity. Once this function was identified, additional statistical data analyses beyond these bioinformatic procedures were conducted using GraphPad Prism 7.01 (GraphPad Software, Inc., La Jolla, CA, USA) and IBM SPSS 25.0 (IBM, Armond, NY, USA). Data distribution within groups was analyzed using the Shapiro–Wilk test, while the presence of outliers was determined using Tukey’s rule. If data showed a normal distribution, a Student’s t-test was performed to assess comparisons between two groups, otherwise, a Mann–Whitney test was used. For comparison between more than two groups, a one-way ANOVA test was applied in the case of one independent variable, and two-way ANOVA when more than two independent variables were considered, followed by uncorrected Fisher’s least significant difference (LSD). Likewise, correlations between the expression levels of *IL-16* and selected genes were conducted using Pearson’s (normally distributed data) or Spearman’s (non-normally distributed data) correlations. The statistical significance threshold for all analyses was established at the two-tailed 5% level (*P*< 0.05).

## 3 Results

### 3.1 IL-16 expression was highest in vWAT from individuals with obesity and was related to inflammatory pathways and serum IL-16 levels were modulated after bariatric surgery

**Figure 1:**
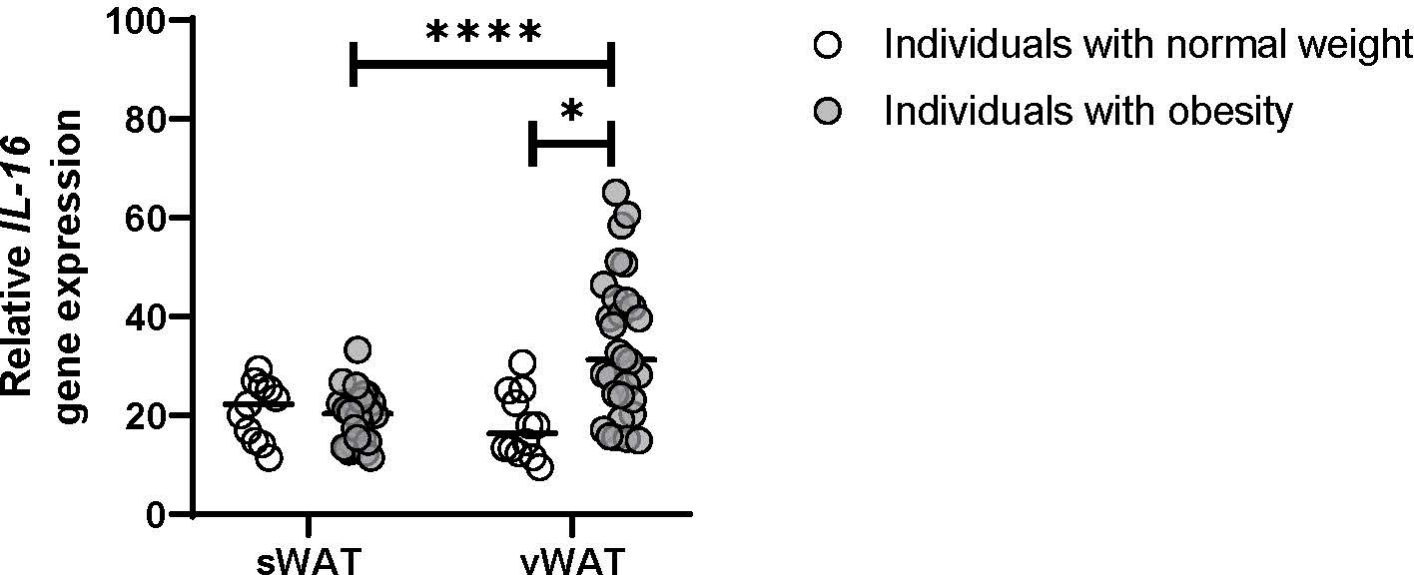
Gene expression of *IL-16* in vWAT and sWAT from individuals with normal weight or obesity (n = 25). Data represent mean ± SEM. Data were analyzed using one-way ANOVA followed by uncorrected Fisher’s least significant difference (LSD). Different letters indicate statistically significant differences at *P*< 0.05 within each compared group. Gene expression was assessed using specific TaqMan probes Hs00913644_m1 for *IL-16* and Hs04194521_s1 for the housekeeping *Ppia* gene. Gene expression was calculated using the 2^-ΔΔCt^ method. *p<0.05, ****p<0.0001.

Individuals with severe obesity showed a higher weight, body mass index (BMI), % hemoglobin A1c, and elevated levels of triglycerides. On the other hand, patients with obesity had lower total cholesterol and HDL-cholesterol concentrations compared with the control group of normal-weight individuals. This suggests that they exhibited obesity-associated metabolic disarray beyond an increase in adiposity (**Table 1**). *IL-16* expression was evaluated in vWAT and sWAT from both groups, finding that *IL-16* expression was increased in vWAT from individuals with obesity compared with sWAT and vWAT from normal-weight individuals (*P<* 0.0001) (**Figure 1**). Higher serum levels of IL-16 were observed in patients with obesity compared with normal-weight individuals, with an increase 6 months after bariatric surgery that is reverted 12 months after the intervention (**Supplementary Figure S1**).

### 3.2 IL-16 gene expression was related to inflammatory and immune cell activation pathways

**Figure 2:**
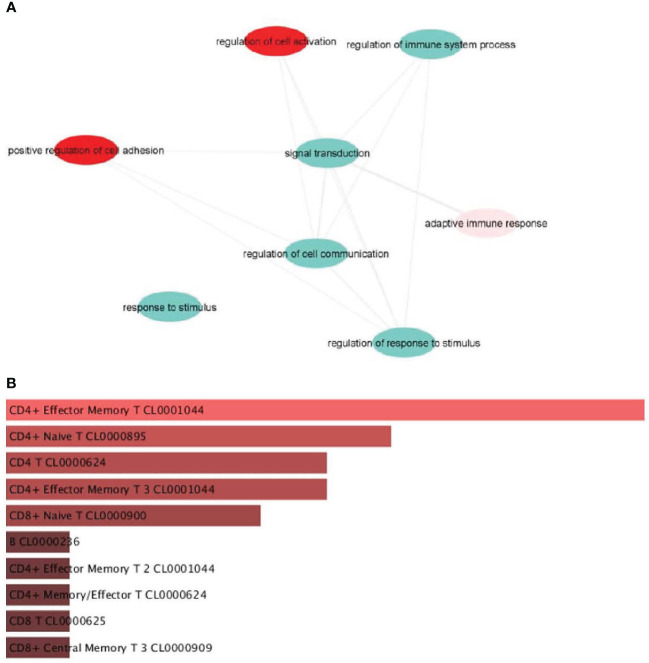
Pathways enriched in genes that correlate with *IL16*. **(A)** Genes positively correlated with *IL16*. **(B)** Identification of the main cell identity of genes correlated with *IL16*.

Public database analysis from our laboratory (GEO repository: GSE236145) showed that *IL-16* is associated with inflammatory processes, including regulation of cell activation, immune system process, adaptive immune response, and regulation of response to stimulus (**Figure 2**). This analysis indicated an association between IL-16 and cell adhesion-positive processes and cell communication (**Figure 2**).

### 3.3 IL-16 altered adipogenesis, fibrosis, and glucose and lipid metabolism during 3T3-L1 differentiation

*In vitro* experiments were performed using 3T3-L1 preadipocytes to evaluate the effect of IL-16 on adipocyte differentiation and the modulation of key markers involved in adipocyte biology. First, 3T3-L1 cell differentiation was evaluated by measuring the accumulation of intracellular lipid droplets and the mRNA expression of adipocyte differentiation markers (**Supplementary Figure S2**). An increase in intracellular lipid droplets at day 7 and elevated levels of genes involved in fat storage, such as Perilipin 1 (*Plin1*) and the adipocyte fatty acid binding protein (*Fabp4*), was observed (*P<* 0.01). Moreover, markers of adipogenesis, such as adiponectin (*AdipoQ*) and peroxisome proliferator-activated receptor gamma (*Pparg*), also increased their expression at day 7 (*P<* 0.01) (**Supplementary Figure S2**).

**Figure 3:**
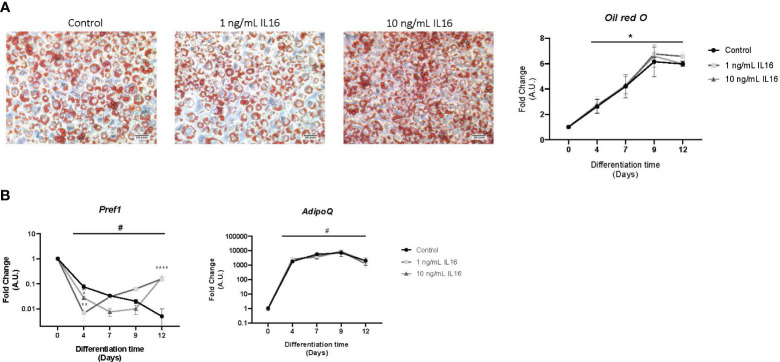
Lipid accumulation and mRNA gene expression in 3T3-L1 cells differentiated in the presence of 1 or 10 ng/mL IL-16. **(A)** Effect of IL-16 on lipid accumulation during adipogenesis (Oil red O staining). **(B)** Effect of IL-16 on the expression of differentiation markers (*Pref1* and *AdipoQ*). Data are represented as relative mRNA levels (arbitrary units (A.U.)), relative to day 0 of differentiation (#p<0,05), and expressed as mean±SEM; n=4. Data were analyzed by one-way ANOVA followed by uncorrected Fisher LSD to compared treatments at the same time. *p<0.05, ****p<0.0001.

Then, 1 or 10 ng/mL of IL-16 was added to the cells to evaluate the effects on adipocyte differentiation *in vitro*. The results showed that IL-16 did not affect the lipid accumulation profile during preadipocyte differentiation (**Figure 3A**). While no changes were seen in *AdipoQ* mRNA expression, incubation with IL-16 induced an increase in *Pref1* expression at day 12, independent of the concentration (1 or 10 ng/mL of recombinant IL-16, *P*< 0.0001) (**Figure 3B**).

### 3.4 IL-16 altered inflammation, glucose and lipid metabolism, and remodeling in mature 3T3-L1 adipocytes

The effect of IL-16 on inflammation, glucose and lipid metabolism, and remodeling, given their involvement in WAT during obesity, was analyzed.

**Figure 4:**
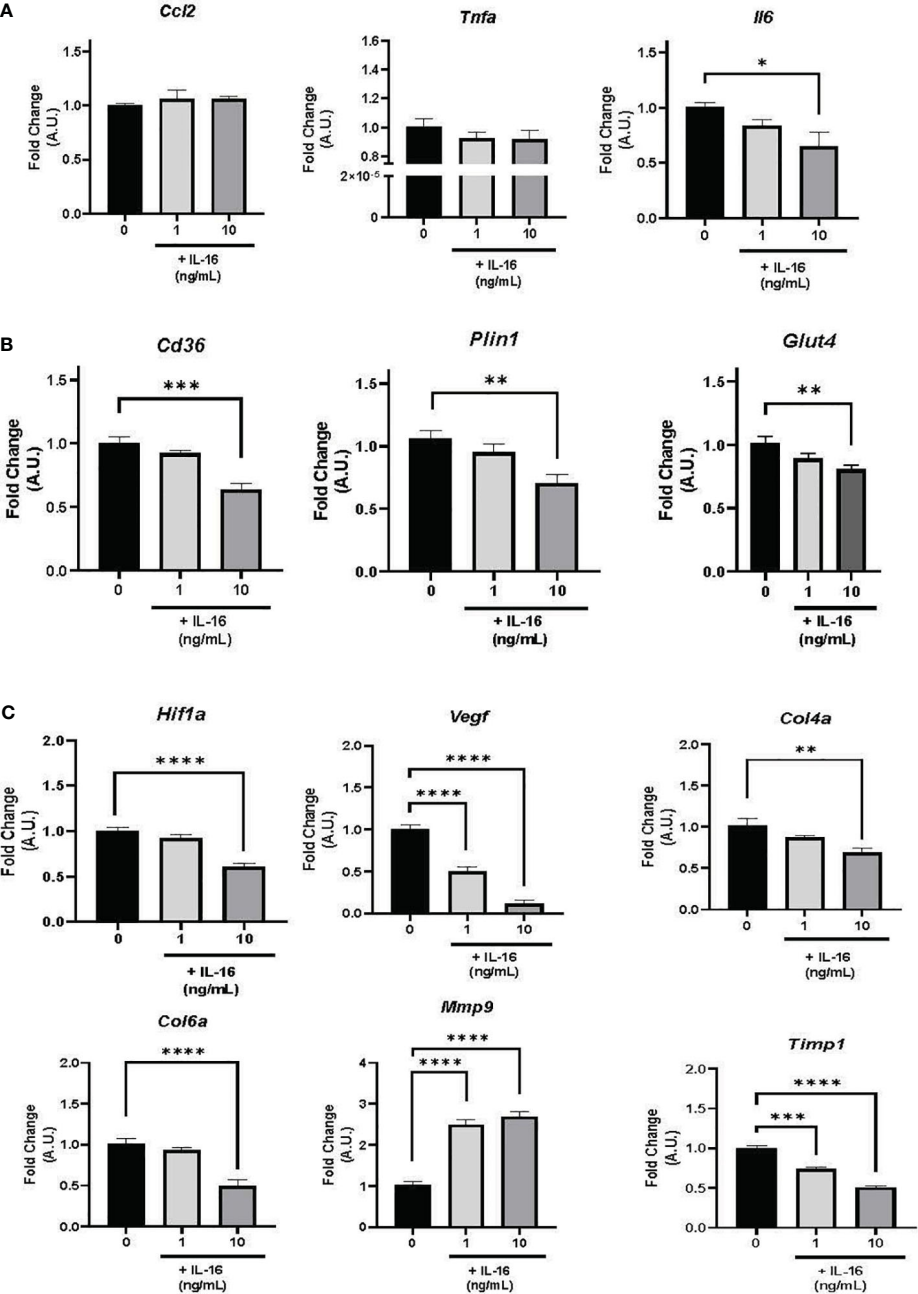
Gene expression in mature 3T3-L1 adipocytes in the presence of 1 or 10 ng/mL IL-16. **(A)** Effect of IL-16 on inflammatory markers (*Ccl2*, *Tnfa*, and *Il6*). **(B)** Effect of IL-16 on genes related to lipid and glucose metabolism (*Cd36, Plin1*, and *Glut4*). **(C)** Effect of IL-16 on remodeling markers (*Hif1a*, *Vegf*, *Col4a*, *Col6a*, *Mmp9*, and *Timp1*). Data represent relative mRNA levels (A.U.) and are expressed as mean ± SEM (n = 8/group). Data were analyzed by one-way ANOVA followed by uncorrected Fisher’s LSD, *P< 0.05, **P< 0.01, ***P< 0.001, ****P< 0.0001.

In terms of inflammation, *Il6* gene expression decreased in response to 10 ng/mL IL-16 (*P*< 0.05), while *Tnfa* and *Ccl2* showed no response to any of the administered doses (**Figure 4A**).

The expression of genes associated with lipid and glucose metabolism (*Plin1, Cd36*, and *Glut4*) was studied, finding that incubation with 10 ng/mL of IL-16 decreased mRNA expression levels of *Plin1* (*P<* 0.01)*, Cd36* (*P<* 0.001), and *Glut4* (*P<* 0.01) (**Figure 4B**).

Finally, incubation with 10 ng/mL of IL-16 decreased the mRNA expression of *Hif1a* (*P*< 0.0001), *Col4a* (*P*< 0.01), and *Col6a* (*P*< 0.0001) (**Figure 4C**); additionally, both 1 and 10 ng/mL of IL-16 reduced the expression of *Vegf* (*P*< 0.0001) (**Figure 4C**). Furthermore, increased expression of the metalloproteinase *Mmp9* and decreased expression of its inhibitor *Timp1* (*P*< 0.0001) were found (**Figure 4C**; **Supplementary Figure S3**).

### 3.5 IL-16 treatment blunted palmitate-induced lipid accumulation, lipolysis, and altered inflammation in mature 3T3-L1 adipocytes

**Figure 5:**
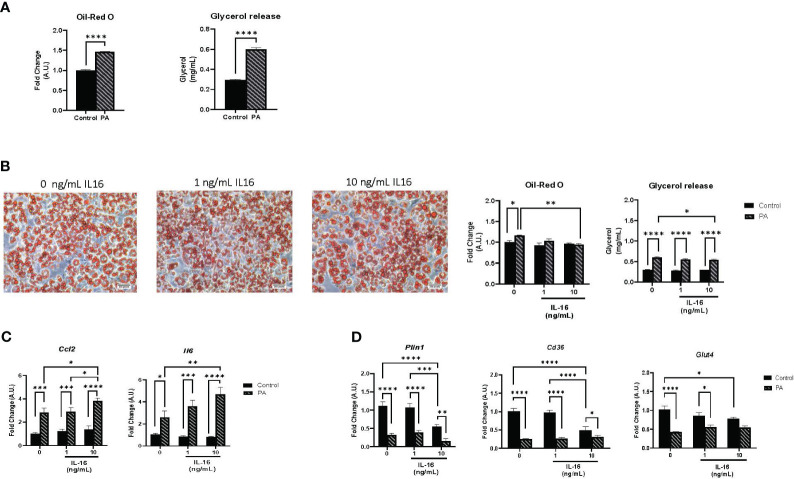
Lipid accumulation, lipolysis, and gene expression in mature 3T3-L1 adipocytes. **(A)** Hypertrophic and lipolytic effects of palmitate treatment on mature adipocytes. Mature 3T3-L1 adipocytes were treated for 24 h with palmitate and lipid accumulation and glycerol secretion were measured. Data represent mean ± SEM (n = 4/group). **(B)** Hypertrophic and lipolytic effects of IL-16 in an obesity context. Mature adipocytes were treated for 24 h with palmitate and IL-16 and lipid accumulation and glycerol secretion were measured. **(C)** Effect of IL-16 on inflammatory markers in palmitate-treated adipocytes (*Ccl2* and *Il6*). Mature 3T3-L1 adipocytes were treated simultaneously with palmitate and increasing doses of IL-16. Data represent mRNA levels (A.U.) relative to the control group and expressed as mean ± SEM (n = 4/group). **(D)** Effect of IL-16 on genes related to lipid and glucose metabolism (*Plin1*, *Cd36*, and *Glut4*). Mature 3T3L1 adipocytes were co-treated with palmitate and increasing doses of IL-16. Data represent mRNA levels (A.U.) relative to the control group and expressed as mean ± SEM (n = 4/group). Data were analyzed by two-way ANOVA followed by uncorrected Fisher’s LSD, *P< 0.05, **P< 0.01, ***P< 0.001, ****P< 0.0001.

Individuals with obesity are reported to have high levels of circulating free fatty acids (FFAs), the molecules most associated with obesity and the development of co-morbidities (22). Palmitate (C16:0) is frequently used in *in vitro* models to enhance adipocyte differentiation and hypertrophy of the mature phenotype (23–25). On day 7, cells were treated with 1 mM palmitate conjugated to BSA for 24 h, according to previous protocols established in our laboratory (21). To corroborate the effect of this treatment, the mRNA expression of different inflammatory markers was measured (**Supplementary Figure S3**). As expected, palmitate treatment promoted an inflammatory pattern, increasing the expression of *Tgfb*, *Ccl2*, and *Il6* (**Supplementary Figure S4**). Next, lipid accumulation and lipolysis were evaluated using Oil red O staining and measurement of secreted glycerol, respectively (**Figure 5**). Palmitate treatment of mature adipocytes increased both lipid accumulation and glycerol release (*P*< 0.0001) (**Figure 5A**).

Mature adipocytes were co-treated with palmitate (1 mM) and IL-16 at concentrations of 1 or 10 ng/mL IL-16 for 24 h to study the impact of IL-16 in the context of obesity. Cells without IL-16 treatment (with and without palmitate) served as controls. The administration of 10 ng/mL IL-16 mitigated the palmitate-induced increase in both lipid accumulation and lipolysis (*P*< 0.05, *P*< 0.01, *P<* 0.0001) (**Figure 5B**).

Regarding inflammation, IL-16, together with palmitate, exerted a synergistic effect enhancing the increase in inflammatory markers *Ccl2* and *Il6* (*P*< 0.05, *P*< 0.01, *P*< 0.001, *P<* 0.0001) (**Figure 5C**). IL-16 treatment at both doses (1 and 10 ng/mL) did not prevent the reduction in *Plin1*, *Cd36*, and *Glut4* caused by palmitate treatment (**Figure 5D**).

## 4 Discussion

Individuals with overweight are reported to have higher IL-16 plasma levels compared with normal-weight individuals; IL-16 levels correlate with body weight, BMI, and waist circumference (26). Additionally, non-obese mice genetically prone to diabetes are protected from developing diabetes when treated with an IL-16 neutralizing antibody (27). In this study, our objective was to elucidate the significance of IL-16 in the context of obesity by examining its levels in a human cohort of patients with obesity and assessing its effect on adipocyte biology. In our cohort, *IL-16* expression was increased in vWAT from individuals with obesity compared with sWAT from individuals with obesity and vWAT and sWAT from normal-weight individuals (**Figure 1**); similarly, obese animal models presented higher levels of IL-16 than lean controls (28). These results suggest that inflammation and lipotoxicity could induce IL-16 secretion in vWAT. Unexpectedly, serum IL-16 levels were increased 6 months after bariatric surgery and then decreased after 12 months to levels similar to those before the surgery. Metabolic surgery has been described to improve systemic glucose and lipid homeostasis (29), however, adipose tissue biology after surgery is still not fully understood. Some metabolic adaptation related to calorie restriction after surgery could be involved in this IL-16 modulation and inflammatory signaling.

vWAT has been linked to an increase in metabolic risk factors (30–32); therefore, identifying novel candidates involved in the adipocyte and immune cell crosstalk that promote metabolic dysregulation would be a turning point in obesity treatment.

Our *in vitro* study showed that adding recombinant IL-16 to undifferentiated 3T3-L1 cells increased *Pref1* expression, suggesting potential adipogenesis impairment. This effect seems to be concentration-independent and was not accompanied by a reduction in *AdipoQ* expression or lipid accumulation.

An impaired adipogenesis capacity and reduced fibrotic signaling in WAT leads to altered lipolysis (33–35), which contributes to the development of metabolic disturbances (36, 37). Lipolysis is regulated by the MAPK/ERK (MEK)1/2 and ERK1/2 pathways (38) and can be activated by *Pref1* (39), leading to the downregulation of perilipins (40). It is tempting to think that IL-16 could play a role in adipogenesis in obesity to promote fibrosis and alter lipid storage in adipocytes.

Next, inflammation in mature adipocytes was examined; IL-16 treatment showed a tendency toward reduced *Tnfa* expression. TNFα is a proinflammatory cytokine whose expression correlates with adiposity in humans and promotes lipolysis in WAT (41). Moreover, a significant reduction in *Il6* was also observed after treatment with 10 ng/mL of IL-16. *Il6* expression was reported to decrease without changes in macrophage infiltration during weight loss (42). Furthermore, *IL6* is regulated by *Tnfa*, and its expression regulates the activity of lipoprotein lipase (43). This observation could be related to a potential effect of IL-16 in inflammatory signaling when inflammation is established, aiming to reduce it and balance the metabolic state.

In terms of lipid and glucose metabolism, the addition of IL-16 produced a reduction in *Plin1* and *Cd36* gene expression, which showed significant results at the dose of 10 ng/mL; reduced *Plin1* expression has been linked to an increased proinflammatory response in WAT from lean mice. Thus, this lipid droplet-binding protein helps to maintain metabolic homeostasis (44), and similarly, adipocyte *Cd36* plays a metabolically protective role (45, 46). Finally, 10 ng/mL IL-16 decreased the expression of the cytokine *Glut4*, suggesting a role in glucose metabolism signaling by impairing glucose uptake.

Taken together, our results indicate that IL-16 may be involved in inflammation, lipid accumulation, and altered glucose signaling, contributing to the development of metabolic diseases.

Subsequently, markers of hypoxia, fibrosis, and WAT remodeling were evaluated; surprisingly, a decrease in *Hif1a* and *Vegf* expression was observed. HIF1α is a transcription factor that contributes to chronic inflammation in obesity (47, 48), and its inhibition in adipocytes leads to reduced fibrosis and inflammation in cell and animal models (49). Moreover, *Vegf* overexpression protects against diet-induced obesity and insulin resistance (50), also, the effect of enhancing VEGF levels on adipose tissue vasculature and the ensuing metabolic phenotypes was reported by AlZaim et al. (51). Thus, a reduction in *Vegf* could be linked to the establishment of obesity and insulin resistance. Therefore, it is tempting to think that IL-16 could be promoting the reduction of *Hif1a*, which causes a decrease in *Vegf* in the context of altered adipose tissue remodeling in obesity. Since changes in genes associated with extracellular matrix (ECM) remodeling were observed, we decided to evaluate the expression of *Mmp9* and its inhibitor, *Timp1*. IL-16 enhanced *Mmp9* and decreased *Timp1* expression, increasing the Mmp9/Timp1 ratio, suggesting that IL-16 could inhibit hypoxia but increase ECM remodeling in WAT.

Adipocytes were treated with 1 mM palmitate to mimic obesity *in vitro*. Palmitate was found to increase the expression of proinflammatory genes, as previously described in the literature (25, 52–55), and promote lipid accumulation and lipolysis. Next, the concomitant effect of adding palmitate and IL-16 was evaluated. The addition of 10 ng/mL IL-16 blunted palmitate-induced lipid accumulation and glycerol release, suggesting that IL-16 may impair lipolysis, leading to adipocyte hypertrophy in an obesity context.

Surprisingly, IL-16 also promoted the expression of *Ccl2* and *Il6* in palmitate-treated adipocytes. Ccl2 is an important factor that promotes macrophage infiltration and lipid transportation in sWAT in animal models (56, 57). On the other hand, in adipocytes, IL-6 induces the release of FFAs and leptin and blunts obesity-associated metabolic complications (58). These data may indicate that IL-16 plays a role in early inflammatory signaling in WAT yet could promote chronic inflammation, leading to altered systemic metabolism.

The evaluation of the effects of IL-16 on lipid and glucose metabolism showed that IL-16 treatment could not fully prevent the reduction in *Plin1* and *Cd36* caused by palmitate; however, adding 10 ng/ml of IL-16 blunted the palmitate-induced decrease in *Glut4* gene expression. These results support the hypothesis that IL-16 may be involved in lipid and glucose metabolism in obesity.

## 5 Limitations

Our work has several limitations. First, as no specific IL-16 receptor is known in adipocytes, it cannot be concluded that the effects of recombinant IL-16 are due to direct signaling. Second, the lack of an *in vivo* model targeting IL-16 makes it difficult to postulate the potential role of IL-16 in systemic metabolism. Third, there was an insufficient number of human WAT samples from biopsies to measure IL-16 protein levels by western blotting. Fourth, owing to the relatively low number of patients with complete clinical data (all women, see **Table 1**), significant correlations were not reported nor could the study be split by gender.

## 6 Conclusion

In conclusion, our results demonstrate that IL-16 is induced in obesity, suggesting its involvement in adipogenesis and cellular homeostasis. This would imply that IL-16 might play a crucial role in maintaining proper adipocyte biology in the context of obesity. Future studies should explore *in vivo* approaches to further evaluate the role of IL-16 in adipose tissue biology in obesity.

## Data availability statement

The original contributions presented in the study are included in the article/**Supplementary Material**. Further inquiries can be directed to the corresponding author/s.

## Ethics statement

The studies involving humans were approved by Institut Germans Trias i Pujol ethical committe (PI8-078 and PI20-351). The studies were conducted in accordance with the local legislation and institutional requirements. The participants provided their written informed consent to participate in this study. Ethical approval was not required for the studies on animals in accordance with the local legislation and institutional requirements because only commercially available established cell lines were used.

## Author contributions

MR-F: Data curation, Investigation, Methodology, Writing – original draft, Formal analysis. PF-G: Formal analysis, Writing – original draft, Software, Writing – review & editing. PC: Writing – review & editing, Supervision. LG: Writing – review & editing. AS-G: Writing – review & editing, Data curation. EM: Data curation, Writing – review & editing. SP: Data curation, Writing – review & editing. JT: Data curation, Writing – review & editing. PM: Data curation, Writing – review & editing. LS: Data curation, Software, Writing – review & editing. GM-G: Writing – review & editing. DS-I: Conceptualization, Data curation, Investigation, Methodology, Project administration, Resources, Supervision, Writing – original draft, Writing – review & editing. LH: Conceptualization, Data curation, Investigation, Methodology, Project administration, Resources, Supervision, Writing – original draft, Writing – review & editing, Funding acquisition, Validation.
