# Supplementary material for: Interleukin-16 is increased in obesity and alters adipogenesis and inflammation in vitro
Source: Front Endocrinol (Lausanne). 2024 Mar 13;15:1346317. doi: 10.3389/fendo.2024.1346317 (PMC10965774; doi:10.3389/fendo.2024.1346317)
Supplement: Supplementary file 1 [file DataSheet_1.doc]

Supplementary Material

**Supplementary figures**

**Figure S1. IL-16 serum levels are modulated after a bariatric surgery.** Serum levels of IL-16 before and after bariatric surgery and comparison with normal weight subject. Data are represented as mean±SEM. Data were analyzed by ordinary one-way ANOVA followed by uncorrected Fisher LSD, different letters indicate statistically significant difference at p<0.05 within each compared group.


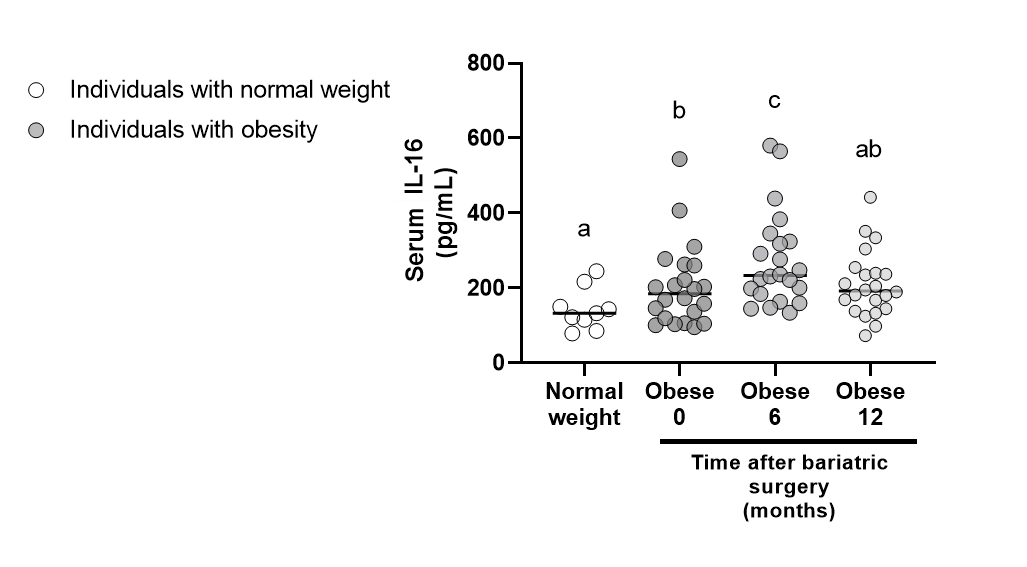


**Figure S2. Lipid accumulation and differentiation markers in 3T3-L1 cells.** (A) Lipid accumulation evaluated by oil red O assay. (B) Relative mRNA expression of *AdipoQ*, *Pref1*, *Pparg*, *Plin*, *Fabp4*, and *Glut4*. Data are represented as Fold Change (arbitrary units (A.U.)), relative to day 0 of differentiation, and expressed as mean±SEM; n=3-4. Data were analyzed by ordinary one-way ANOVA followed by uncorrected Fisher LSD, *p<0.05, **p<0.01, ***p<0.001, ****p<0.0001.

1. B)

**Figure S3. *Mmp9/Timp1* ratio.** 3T3-L1 mature adipocytes were treated with increasing doses of IL-16 (1 and 10 ng/mL). Data are represented as relative mRNA level [arbitrary units (A.U.)], relative to 0 ng/mL IL-16 and expressed as mean ± SEM (n = 4). Data were analysed by one-way ANOVA followed by uncorrected Fisher’s LSD, *****P* < 0.0001.

**Figure S4**. mRNA gene expression of inflammatory and hypertrophic markers on adipocytes treated with palmitate. Mature adipocytes were treated for 24 h with palmitate and gene expression was measured. Data represent mean ± SEM (n = 4/group). Data were analyzed by unpaired *t* test. *****P* < 0.0001.
