# Supplementary material for: Interleukin-16 is increased in obesity and alters adipogenesis and inflammation in vitro
Source: Front Endocrinol (Lausanne). 2024 Mar 13;15:1346317. doi: 10.3389/fendo.2024.1346317 (PMC10965774; doi:10.3389/fendo.2024.1346317)
Supplement: Supplementary file 2 [file Table_1.docx]

| **Supplementary Table S1:** 3T3-L1 culture media.   \| **Medium** \| **Reagent** \| **Reference** \| **Final concentration** \| \| --- \| --- \| --- \| --- \| \| Growth medium \| DMEM \| Gilco^TM^, 41966-029 \|  \| \| Newborn calf serum (CBS) \| Gilco^TM^, 1803780 \| 10% \| \| Penicillin–streptomycin antibiotics (P/S) \| Sigma-Aldrich, 15140-122 \| 1% \| \| Maintenance medium \| DMEM \| Gilco^TM^, 41966-029 \|  \| \| Fetal bovine serum \| Gilco^TM^, 10270-106 \| 10% \| \| Penicillin-streptomycin antibiotics (P/S) \| Sigma-Aldrich, 15140-122 \| 1% \| \| Induction medium \| Maintenance medium \|  \|  \| \| Insulin \| Sigma-Aldrich, I9278 \| 10 µg/mL \| \| Dexamethasone \| Sigma-Aldrich, D4902 \| 0.25 mM \| \| IBMX \| Sigma-Aldrich, I7018 \| 500 µM \| \| Differentiation medium \| Maintenance medium \|  \|  \| \| Insulin \| Sigma-Aldrich, I9278 \| 10 µg/mL \| \| Freezing medium \| CBS \| GibcoTM, 1803780 \| 90% \| \| DMSO \| Sigma-Aldrich, D2438 \| 10% \|   DMEM, Dulbecco’s modified eagle medium; DMSO, dimethyl sulfoxide; IBMX, 3-isobutyl-1-methylxanthine. |
| --- | --- | --- | --- | --- | --- | --- | --- | --- | --- | --- | --- | --- | --- | --- | --- | --- | --- | --- | --- | --- | --- | --- | --- | --- | --- | --- | --- | --- | --- | --- | --- | --- | --- | --- | --- | --- | --- | --- | --- | --- | --- | --- | --- | --- | --- | --- | --- | --- | --- | --- | --- |
