# Supplementary material for: Interleukin-16 is increased in obesity and alters adipogenesis and inflammation in vitro
Source: Front Endocrinol (Lausanne). 2024 Mar 13;15:1346317. doi: 10.3389/fendo.2024.1346317 (PMC10965774; doi:10.3389/fendo.2024.1346317)
Supplement: Supplementary file 3 [file Table_2.docx]

| **Supplementary Table S2:** Primers used for qPCR gene expression analyses in 3T3-L1 adipocytes.   \| **Gene Symbol** \| **Gene Name** \| **Forward primer** \| **Reverse primer** \| \| --- \| --- \| --- \| --- \| \| *Adipoq* \| Adiponectin \| GCAGAGATGGCACTCCTGGA \| CCCTTCAGCTCCTGTCATTCC \| \| *Ccl2* \| C–C motif chemokine ligand 2 \| GCAGGTGTCCCAAAGAAGCT \| CAGCACAGACCTCTCTCTTGA \| \| *Cd36* \| CD36 molecule \| TTGTACCTATACTGTGGCTAAATGAGA \| CTTGTGTTTTGAACATTTCTGCTT \| \| *Col4a1* \| Collagen type IV alpha 1 chain \| TTAAAGGACTCCAGGGACCAC \| CCCACTGAGCCTGTCACAC \| \| *Col6a1* \| Collagen type VI alpha 1 chain \| GTCCACGTGCTCTTGCATC \| GCAAGGATGAGCTGGTCAA \| \| *Fabp4* \| Fatty acid binding protein 4 \| GGATGGAAAGTCGACCACAA \| TGGAAGTCACGCCTTTCATA \| \| *Glut4* \| Glucose transporter type 4 \| GATGACCGTGGCTCTGCT \| GCTCTGCCACAATGAACCA \| \| *Hif1a* \| Hypoxia-inducible factor-1 \| GCACTAGACAAAGTTCACCTGAGA \| CGCTATCCACATCAAAGCAA \| \| *Il6* \| Interleukin 6 \| GATGGATGCTACCAAACTG \| CCAGGTAGCTATGGTACTCCAGAA \| \| *Lep* \| Leptin \| AGAAGATCCCAGGGAGGAAA \| TGATGAGGGTTTTGGTGTCA \| \| *Mmp9* \| Matrix metallopeptidase 9 \| ACGACATAGACGGCATCCA \| GCTGTGGTTCAGTTGTGGTG \| \| *Plin1* \| Perilipin 1 \| AACGTGGTAGACACTGTGGTACA \| TCTCGGAATTCGCTCTCG \| \| *Pparg* \| Peroxisome proliferator activated receptor gamma \| CGCTGATGCACTGCCTATGA \| AGAGGTCCACAGAGCTGATTCC \| \| *Ppia* \| Peptidyl-prolyl cis-trans isomerase A \| TGCCAAGACTGAATGGCTG \| ATTCCTGGACCCAAAACGCT \| \| *Pref1* \| Preadipocyte factor 1 \| CGGGAAATTCTGCGAAATAG \| TGTGCAGGAGCATTCGTACT \| \| *Tgfb* \| Transforming growth factor beta \|  \|  \| \| *Timp1* \| Tissue inhibitor of metalloprotease-1 \| GCAAAGAGCTTTCTCAAAGACC \| AGGGATAGATAAACAGGGAAACACT \| \| *Tnfa* \| Tumor necrosis factor alpha \| CTGTAGCCCACGTCGTAGC \| TTTGAGATCCATGCCGTTG \| \| *Vegf* \| Vascular endothelial growth factor A \| AAGACAGAACAAAGCCAGAAAA \| AGAGGTCTGGTTCCCGAAA \| |
| --- | --- | --- | --- | --- | --- | --- | --- | --- | --- | --- | --- | --- | --- | --- | --- | --- | --- | --- | --- | --- | --- | --- | --- | --- | --- | --- | --- | --- | --- | --- | --- | --- | --- | --- | --- | --- | --- | --- | --- | --- | --- | --- | --- | --- | --- | --- | --- | --- | --- | --- | --- | --- | --- | --- | --- | --- | --- | --- | --- | --- | --- | --- | --- | --- | --- | --- | --- | --- | --- | --- | --- | --- | --- | --- | --- | --- | --- | --- | --- | --- |
